# Wound Healing Enhancement and Physical Characterization of Bioadhesive Poly(acrylic acid)/Polyvinylpyrrolidone Complex Gels

**DOI:** 10.3390/gels11040300

**Published:** 2025-04-19

**Authors:** Ayaka Oouchi, Tomoko Ito, Yasuhiro Katahira, Hideaki Hasegawa, Kenichi Nakamura, Izuru Mizoguchi, Takayuki Yoshimoto, Yoshiyuki Koyama

**Affiliations:** 1 Kawasaki Frontiens R&D Center, Toagosei Co., Ltd., Kanagawa 210-0821, Japan; ayaka_oouchi@mail.toagosei.co.jp (A.O.); kenichi_nakamura@mail.toagosei.co.jp (K.N.); 2 Institute of Medical Science, Tokyo Medical University, Tokyo 160-8402, Japan; yasuhiro@tokyo-med.ac.jp (Y.K.); h_hasega@tokyo-med.ac.jp (H.H.); imizo@tokyo-med.ac.jp (I.M.); yoshimot@tokyo-med.ac.jp (T.Y.); 3 Obara Hospital Research Institute, Tokyo 164-0012, Japan

**Keywords:** wound healing enhancement, bioadhesion, poly(acrylic acid), polyvinylpyrrolidone, hydrogel

## Abstract

In addition to protection against microorganisms and hemostasis, wound dressings are now expected to actively promote healing. A water-absorbing complex of poly(acrylic acid) (PAA) and polyvinylpyrrolidone (PVP) was developed by mixing the polymers under specific conditions. This complex swells in water and adheres strongly to biological tissues. Upon application to a wound, it absorbs blood, swells, and adheres firmly, providing coverage. During this process, blood cells that infiltrate the gel secrete growth factors and other bioactive molecules, which are retained and gradually released toward the wound, promoting healing. In the present study, the mechanical properties of the PAA/PVP complexes were analyzed, and their healing-promoting effects were examined. In a diabetic mouse skin wound model, untreated wounds remained over 95% of their original size after 4 days. In contrast, wounds treated with the PAA/PVP complex shrank to 70–75% of their original size by day 4, and further reduced to 17–23% by day 11. Histological analysis on day 11 showed complete or nearly complete re-epithelialization in PAA/PVP-treated wounds, while untreated wounds exhibited incomplete tissue regeneration. These results suggest that the PAA/PVP complex not only provides physical protection, but also facilitates tissue repair, demonstrating its potential as a next-generation wound dressing.

## 1. Introduction

Dressing is an essential method for wound care. Various types of dressings suitable for various wounds have been extensively researched and developed, and many types of dressings are currently available on the market [1]. One of the major roles of wound dressings is to act as a barrier in place of the skin to prevent the invasion of infectious microorganisms [2]. Since Louis Pasteur showed that these living microorganisms come from outside, rather than spontaneously being generated within, the body in the 19th century, the skin has been thought to act as a physical barrier against the invasion of pathogens, and damaged areas of skin are regarded as being at risk of infection. Another important role of these dressings is to protect against external stimuli. Since the nerve endings are exposed at the wound site, contact with air, clothing, etc., causes pain. A hydrogel-like wound dressing softly envelops the nerve endings to reduce pain [3]. Hemostasis is also one of the primary roles of wound dressings. When a blood vessel breaks and starts to bleed, hemostasis occurs in the body to prevent excess blood loss during an injury. Blood coagulation starts immediately to form a clot. Temporary local vasoconstriction is also induced to reduce blood flow to the injury site. This is a natural hemostatic process that forms a thrombus and stops bleeding. When wounds are too large to stop bleeding naturally, in cases such as severe trauma, surgery, and drug-induced coagulopathies, topical wound dressings have been used to control hemorrhage and assist wound healing [4].

In recent years, the goal of wound dressings has expanded beyond protection and hemostasis to include actively accelerating the healing process. Wound healing is a complex, multi-phase process involving inflammation, proliferation, and remodeling [5]. The first stage of wound healing is hemostasis and the formation of a provisional wound matrix. The inflammatory process almost simultaneously gets activated with the recruitment of neutrophils and monocytes, and later by macrophages [5,6]. The inflammatory phase is activated and regulated by multiple cytokines and chemokines released from the cells at the wound site. Neutrophils and monocytes produce proinflammatory cytokine IL-6, which is an important factor in initiating the healing response. Those cells also produce IL-1, along with macrophages [7].

The wound repair process then shifts, overlapping with the inflammatory phase, towards the proliferative phase, and finally to a remodeling phase. Platelets are essential, not only in forming a fibrin clot to stop bleeding, but also to secrete a growth factor, platelet-derived growth factor (PDGF), the most potent mitogen for fibroblasts. Then, the fibroblasts activated and recruited by PDGF migrate to the wound site and begin to proliferate and secrete various growth factors, such as epidermal growth factor (EGF), fibroblast growth factor (FGF), transforming growth factor beta (TGF-b), and vascular endothelial growth factor (VEGF) families, which are of importance in angiogenesis and in constructing a new extra-cellular matrix scaffold [8]. These processes are conducted by cells and cytokines or growth factors. A moist environment is preferred for faster migration of the cells and the proteins. It is also desirable for the prevention of cell death, and for facilitating the interaction of growth factors with their target cells. The statement that a closed wound heals faster than an open wound is found in papyrus written over 3600 years ago, where occlusive wound dressing was recommended. From the mid-1900s, the effect of moisture on wound repair began to be reported in the literature [9]. In 1962, in a pig study, Winter et al. found that wet wounds epithelialized faster than dry wounds [10]. Hinman and Maibach demonstrated that an occlusive dressing was also effective in humans [11].

Disadvantages have also been advocated, such as the fact that excessive moisture may cause the growth of bacteria and fungi. Fluid drained from the wound is rich in proteolytic enzymes, which harm the normal skin cells around the wound. Nevertheless, today, “moisture healing”, which provides conditions suitable for wound healing by controlling the humidity of the wound site environment, has become a representative method of promoting healing [9]. Since 1971, when the first film dressings made of the polyurethane film and hydrocolloid composite came onto the market, various types of moisture wound dressings, such as hydrocolloids, hydrogels, semipermeable film, foam, and alginates, have already come onto the market.

In addition to the above properties, wound dressings are required to have the following properties: (1) to absorb or remove excess exudate, (2) be easily removed or replaced, (3) biocompatible, (4) cost-effective, (5) provide esthetic comfort, and sometimes be required to have high gas permeability, elasticity, and biodegradability [1]. In an effort to meet these requirements, various types and compositions of hydrogels have been developed as materials for wound dressings [12,13]. However, an ideal dressing that satisfies all of the necessary requirements has yet to be developed. Among these requirements, tissue adhesiveness is especially important. Hydrogels that lack intrinsic adhesion require external adhesives or bandages, which can irritate the skin or compromise comfort during prolonged use. Adequate adhesive strength is necessary not only to ensure that the dressing remains securely in place, but also to facilitate precise and stable application. However, in terms of handling during application, strong adhesion to surgical tools or gloves impairs usability. Therefore, an ideal hydrogel should adhere firmly and instantly to moist tissue while remaining non-sticky to dry instruments.

In addition, from the perspective of removal, the dressing must not interfere with the wound healing process by causing pain or tissue damage during detachment. Consequently, it is important that the hydrogel can be removed with minimal stimulus when necessary. To address this, several approaches have been developed, such as temperature-responsive protein/polyphenol complexes that can be easily detached by placing an ice bag on the surface of the hydrogel [14]. A composite hydrogel named PBOF comprising polyvinyl alcohol, borax, oligomeric procyanidin, and ferric ion was designed as a multifunctional wound dressing which disassembles in water [15]. However, the preparation of such hydrogels typically requires specialized materials and complicated procedures, which pose challenges for practical and widespread clinical use.

In previous studies [16,17,18], a highly swollen hydrogel composed of poly(acrylic acid) (PAA) and poly(vinylpyrrolidone) (PVP) was developed via a unique solid–liquid interface process. After freeze-drying, the resulting PAA/PVP sponge re-swelled rapidly in water, forming a soft, biocompatible gel with strong adhesion to biological tissues. Moreover, the swellable PAA/PVP complex exhibits distinctive handling characteristics that differentiate it from other hydrogel dressings. In its dry state, the complex does not adhere to tissues, metal instruments, or gloves, enabling precise and stress-free application. Upon contact with moist biological tissue, however, it adheres rapidly and strongly, providing stable fixation throughout the usage period without displacement or peeling. Importantly, the gel can be removed with minimal irritation: if the wound has largely healed and begun to dry, the gel also dries and detaches easily; if the wound remains moist, the gel can be gently washed away with a large volume of water, minimizing the risk of secondary injury during removal.

In addition to these practical handling benefits, this material demonstrated high hemostatic efficacy even in patients receiving antithrombotic therapy [18]. In clinical dental applications, this PAA/PVP sponge achieved immediate hemostasis after tooth extraction and exhibited favorable handling. Notably, the treated wounds appeared to heal more rapidly and cleanly than those managed with conventional gauze. It was hypothesized that absorbed blood components, including platelets and leukocytes, release cytokines and growth factors that are retained within the gel and gradually delivered to the wound surface, contributing to enhanced healing.

In this study, it was newly discovered that the swelling-type PAA/PVP complex gel, in addition to its previously reported strong tissue adhesion and hemostatic effects, also promotes wound healing. Moreover, our experimental results suggest that this healing-promoting effect is not solely due to the maintenance of a moist environment, but also involves the retention of cytokines secreted from blood-derived cells within the gel, followed by their gradual release toward the wound site.

This study aims to evaluate the wound-healing efficacy of the PAA/PVP complex gel using a diabetic mouse skin defect model, and to characterize the mechanical and biological properties of the material that may contribute to its performance. These findings suggest a unique combination of practical utility and biological functionality that may distinguish this dressing from conventional hydrogel-based materials.

## 2. Results and Discussion

Although PAA and PVP do not chemically react with each other, it has been reported that when their aqueous solutions are mixed, they immediately form a polymer complex through hydrogen bonding between the carboxyl groups of PAA and the pyrrolidone rings of PVP [19]. In addition, hydrophobic interactions between the polymer backbones can lead to the formation of water-insoluble aggregates and precipitation [20]. The formation of these hydrophobic interactions requires extensive surface contact between the hydrophobic regions of the polymer chains. To achieve this, the polymer backbones must be sufficiently mobile, allowing them to rearrange and align into a zipper-like configuration through sliding and shifting. It was hypothesized that restricting the free mobility of one of the polymer chains could prevent the formation of the zipper-like structure and suppress hydrophobic interactions, thereby enabling the formation of a water-swellable PAA/PVP complex.

Based on this hypothesis, a solid–liquid interface method was devised to induce complex formation. In the solid/liquid interfacial complexation method—where solid PAA is brought into contact with an aqueous PVP solution—the motion of the PAA chains is significantly restricted by the entanglement effects inherent in the semi-solid, swelling state. This restriction prevents the formation of the zipper-like alignment, thereby suppressing hydrophobic associations and allowing the formation of a water-swellable complex gel [16]. In this process, an aqueous solution of PAA was first dried in a container to form a solid PAA film. An aqueous PVP solution was then added onto the film, allowing it to swell and form the complex. The resulting material was subsequently freeze-dried to obtain the sponge-like complex. Upon the addition of water, the dried sponge re-swelled immediately, forming a hydrated gel with strong adhesion to biological tissues and demonstrating excellent potential for wound dressing applications. The basic properties of the PAA/PVP complex gel, including its swelling behavior, biodissolvability, and cytocompatibility, were thoroughly characterized in our previous study [17]. In brief, the gel exhibits rapid swelling behavior upon hydration, and shows minimal cytotoxicity in the WST-1 assay. These properties make it suitable for biomedical applications such as wound care.

Initially, the effect of the swelling time of the PAA film in a PVP solution on the strength of the resulting sponge was investigated by three-point bending strength measurements. The composition of ingredients was selected based on both the previous literature and our own prior findings. It has long been reported that poly(acrylic acid) (PAA) and polyvinylpyrrolidone (PVP) form interpolymer complexes most efficiently when the repeating units of the two polymers are present at a 1:1 ratio [19]. Consistent with this, our previous studies demonstrated that, under the preparation method used in this study, the complex prepared at a PAA:PVP ratio of 1:1 (in repeating units; 1:1.54 by weight) exhibited the highest mechanical strength under wet conditions [16]. Therefore, this ratio was adopted for the present work. Regarding hyaluronic acid (HA), our earlier research showed that adding 20 wt% HA relative to PAA significantly improved the mechanical strength of the swollen gel without compromising its adhesive properties [18]. Based on these findings, HA was incorporated at a weight ratio of 20% relative to PAA in this study as well. In the present study, a fixed concentration of PVP was used for preparing the PAA/PVP complex. Although the PVP concentration affects the volume and density of the lyophilized solid complex, it has minimal impact on the adhesive properties after rehydration, since the complex becomes a hydrogel whose adhesion depends primarily on the amount of absorbed water. Stick-shaped PAA/PVP sponges containing HA were then prepared at PAA:PVP:HA = 1:1.54:0.2 in weight at swelling times of 0 min (freeze-dried soon after the addition of PVP solution), 20 min, 60 min, and 20 h, and a three-point bending test was conducted.

**Figure 1:**
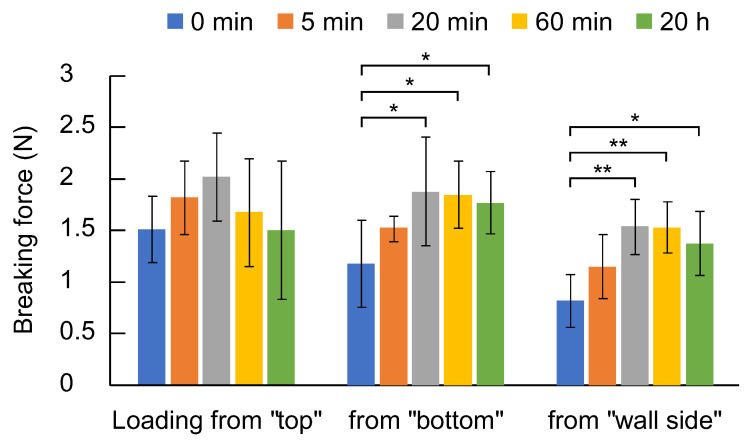
Force required to break stick-shaped PAA/PVP sponges (PAA:PVP:HA = 1:1.54:0.2 by weight) prepared at different swelling times (0 min, 5 min, 20 min, 60 min, or 20 h), evaluated by a three-point bending test. The load was applied from the bottom, top, or wall side (*n* = 5, mean ± SD, * *p* < 0.05, ** *p* < 0.01).

To distinguish each surface of the sponge, the surface that was attached to the bottom of the container when prepared is referred to as the “bottom”, and the surface that was facing upwards is the “top”. The side that was in contact with the container wall when prepared is referred to as the “wall side”. Figure 1 shows the force required to break the sponge when a load was applied from the bottom, top, or wall side. Although no significant difference in the strength was observed, the samples prepared with swelling times of 20 min exhibited the highest strength in terms of average values. When the force was applied from the “bottom” or “ wall side “, the breaking strength of the samples prepared with a swelling time of 20 min or longer was significantly higher than that of those with a swelling time of 0 min.

**Figure 2:**
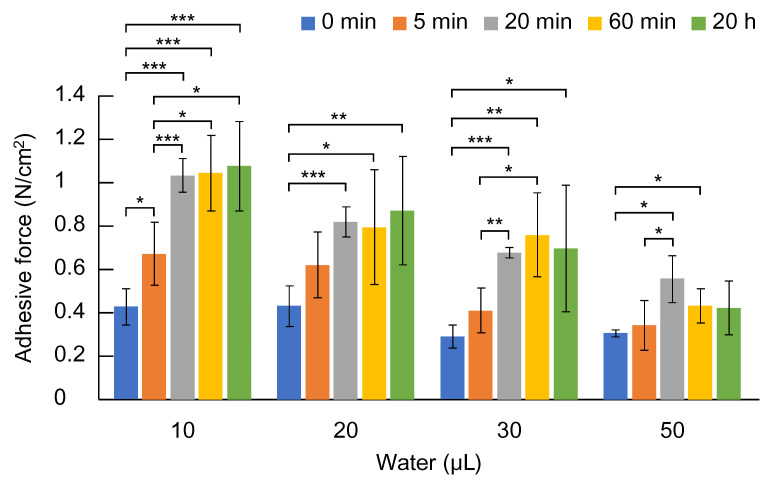
Pull-off adhesive strength of PAA/PVP complexes prepared with different swelling times (0 min, 5 min, 20 min, 60 min, or 20 h) on synthetic leather wetted with varying amounts of water (10, 20, 30, or 50 μL) (*n* ≧ 4, mean ± SD, * *p* < 0.05, ** *p* < 0.01, *** *p* < 0.001).

Next, the pull-off adhesive strength of sponge-like PAA/PVP complexes prepared with various swelling times to wet synthetic leather was compared. PAA/PVP sponge samples were prepared at PAA:PVP:HA = 1:1.54:0.2 in weight. Although the adhesion strength of PAA/PVP complex gels can be influenced by the mixing ratio of the two polymers, the relationship is not straightforward in our system. It was found that a PAA-to-PVP repeating unit ratio of approximately 1:1 yielded the highest average adhesion strength. However, because our complex is formed by allowing a PVP solution to penetrate a pre-solidified PAA layer, the composition within a single sample is not homogeneous [18]. Local variations result in regions that are relatively PAA-rich or PVP-rich, leading to sample-to-sample variability. Consequently, while the 1:1 ratio tends to perform best on average, the differences were not statistically significant due to large standard deviations, and were therefore not emphasized in the present study. A synthetic leather was wetted by a given amount of water. Stick-type complex sponges were cut, and the cut surface was attached to the wetted leather. Regardless of the swelling time during sample preparation, the force required for detachment tended to decrease as the amount of added water increased (Figure 2). The samples prepared with a swelling time of 20 min or longer exhibited higher adhesion strength to the wet synthetic leather compared to those with swelling times of 0 and 5 min, with significant differences observed in many cases. On the other hand, no significant differences were observed among the samples prepared with swelling times of 20 min, 60 min, and 20 h, showing almost the same adhesion strength. As previously reported in our earlier study [17], an excessively long swelling time may cause hydrophobicity and phase separation of PAA/PVP complexes under certain conditions. In this study, a water-swellable PAA/PVP complex sponge was prepared using the solid/solution interface complexation method, which restricts the formation of zipper-like alignment and thereby suppresses hydrophobic association between the polymer chains. However, since the suppression of hydrophobic interactions relies solely on the physical entanglement of the polymer chains, prolonged contact under certain conditions may still allow some hydrophobic bonding to occur. This can result in partial precipitation or turbidity within the swollen gel over time. Therefore, in subsequent experiments, the swelling time was set to 20 min.

**Figure 3:**
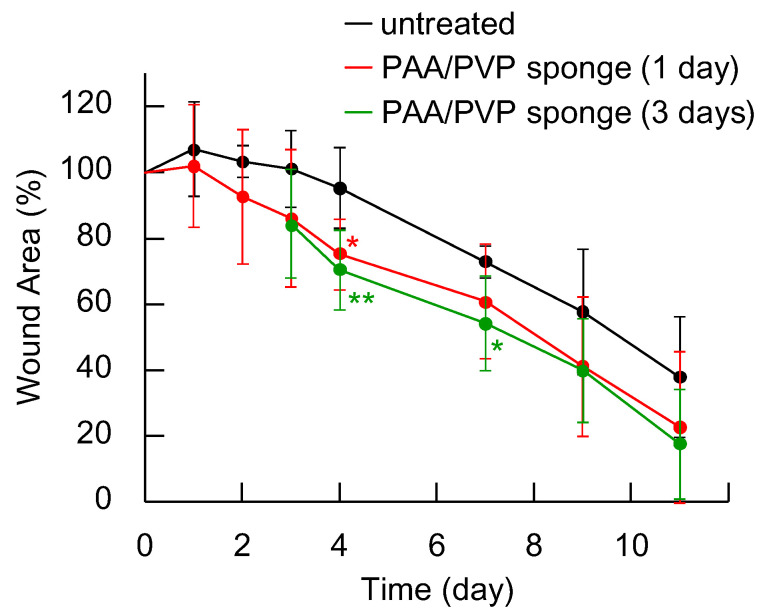
Wound area of mice in the untreated group and the PAA/PVP sponge-treated groups (1 day and 3 days) (*n* = 6, mean ± SD, * *p* < 0.05, ** *p* < 0.01; red asterisk: between PAA/PVP sponge (1 day) and untreated; green asterisk: PAA/PVP sponge (3 day) and untreated).

In our earlier studies [16,17,18], it was reported that the PAA/PVP complex exhibited high hemostatic efficiency not only in animal studies, but also in clinical studies after dialysis treatment or tooth extraction, even in patients on antithrombotic drugs. Moreover, rapid and clean healing was often observed in the patients treated with the PAA/PVP complex sponges. As mentioned in the Introduction, wound dressings are increasingly expected to possess the ability to accelerate wound healing. When the dried PAA/PVP sponge is placed on the bleeding wound site, it rapidly absorbs the blood and body fluid to form an adhesive hydrogel. Then, it covers the wound zone to create a sufficient moist humid atmosphere, which is preferred for wound healing processes. Moreover, the PAA/PVP complex swollen by the blood contains platelet and leukocytes, which release various cytokines and growth factors. The swollen gel is expected to encapsulate those proteins and release slowly toward the wound. Therefore, covering the wound with a PAA/PVP complex is expected to efficiently promote wound healing. The healing-promoting effects of the PAA/PVP complex sponge applied to the wound were then examined in diabetes-model mice. The biocompatibility and cytotoxicity of the PAA/PVP complex gel used in this study have been comprehensively evaluated in our previous work, including both in vitro assessments and clinical investigations [17,18]. Since the formulation and preparation protocol of the complex are identical to those previously reported, no additional biocompatibility assessment was conducted in this study. Nevertheless, its favorable safety profile, including in diabetic wound conditions, is further supported by the absence of adverse tissue responses observed in the present in vivo experiments. Two full thickness-round skin wounds (8 mm in diameter) were made on the back of each db/db mouse, and we divided them into three groups: Group 1, labeled “untreated”, served as the control with no treatment. In Group 2, labeled “PAA/PVP sponge (1 day)”, the wound surface was covered by applying the sample directly, which was removed after one day. In Group 3, labeled “PAA/PVP sponge (3 days)”, the sample was similarly applied to the wound and removed after three days. The wound size was measured up to day 11. Blood samples were collected from one mouse in each group on day 7, and blood glucose levels were measured. The results ranged from 18 to 32 mmol/L, confirming a diabetic state in all cases. As shown in Figure 3, in untreated mice, the wound area remained over 95% of its original size after 4 days. In contrast, wounds covered with the PAA/PVP complex sponge, whether for one day or three days, showed significantly faster reduction and shrank to 70–75% of their original size on day 4. The wounds covered for 1 or 3 days showed rapid healing also in the later stages, shrinking to 17–23% of their original size by day 11.

**Figure 4:**
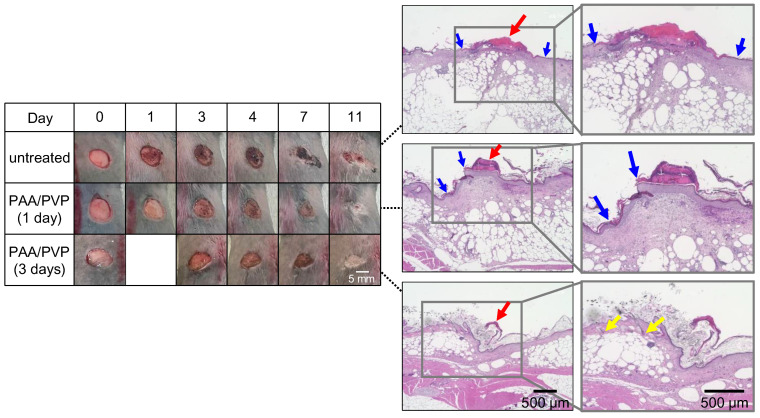
Images of wounds from mice in the untreated group, and the PAA/PVP sponge-treated groups (1 day and 3 days), from day 0 to day 11, and microscope images of HE staining tissue samples collected on day 11. Red, blue, and yellow arrows indicate scabs, the edge of epithelialization, and hair follicles accompanied by sebaceous glands, respectively. Scale bar = 500 µm.

To evaluate tissue regeneration, tissue samples surrounding the wounds were collected on day 11 post-injury, stained with hematoxylin and eosin (HE), and observed under a microscope. Representative images are shown in Figure 4. The histological findings demonstrated notable differences in tissue regeneration between groups. In the untreated group, some degree of re-epithelialization was observed; however, granulation tissue was absent beneath the scab in three out of six samples, indicating incomplete tissue regeneration. Hair follicles and sebaceous glands were either absent or poorly defined, and the dermal and subcutaneous layers exhibited a disorganized structure. In contrast, the group in which the PAA/PVP sponge was applied and removed after one day showed nearly complete re-epithelialization in all six samples, although one sample exhibited a small area with insufficient granulation tissue formation. Notably, in the group treated for three days, complete re-epithelialization was achieved in all samples, and the dermal and subcutaneous layers were well organized. Multiple structures of hair follicles accompanied by sebaceous glands were observed, indicating advanced wound healing. These findings further support the wound-healing efficacy of the PAA/PVP sponge.

The PAA/PVP complex sponge was not covered with something like a waterproof sheet, so moisture could evaporate. However, even after three days, the sponge remained hydrated, likely due to exuded bodily fluids, and the covered wounds retained moisture. This moist environment created by the sponge may have accelerated the wound healing process. On the other hand, continued coverage with dressings that have poor moisture permeability has recently been reported to cause skin maceration due to excessive exudate, not only inducing discomfort and pain, but also potentially delaying the healing process. Skin hyper-hydration may penetrate deeper layers of the skin than previously thought, causing more extensive damage and prolonging the treatment period, highlighting the importance of proper exudate management [21]. Therefore, dressings with absorbent and moisture-evaporating properties that match the amount of exudate are desirable. The PAA/PVP sponge is expected to prevent such adverse effects of maceration, as the hydrogel formed by the absorbed water swells, absorbing exudate while simultaneously allowing moisture to evaporate at a moderate rate. Although no direct comparison was made with commercial wound dressings in this study, many marketed products are designed to be waterproof and prevent moisture evaporation. While this may help retain hydration, it also increases the risk of excessive exudate accumulation and subsequent maceration. In contrast, the PAA/PVP sponge maintains a more balanced hydration environment by allowing gradual evaporation, potentially reducing the risk of skin maceration while still supporting wound healing.

Another possible reason for the accelerated healing is that the hydrated complex retained cytokines such as VEGF within its structure, which were either exudated from the wound or secreted by platelets infiltrated into the complex during blood-induced swelling, and slowly released them toward the wound surface. To evaluate this behavior, an in vitro release experiment was conducted. The PAA/PVP sponge was applied to the wound overnight. Afterward, the sponge swollen by blood and body fluid was placed on an agarose gel, and VEGF transferred into the agarose gel was measured by ELISA.

**Figure 5:**
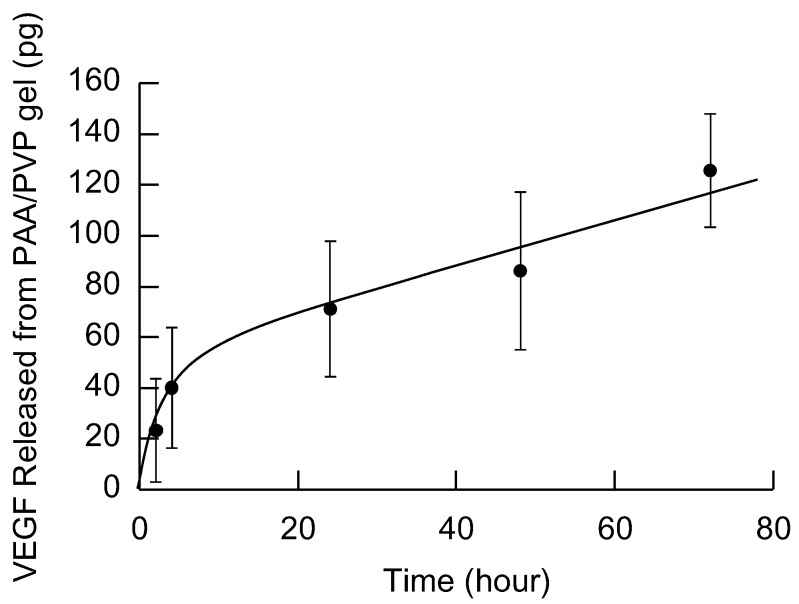
Amount of VEGF transferred into the agarose gel from the PAA/PVP sponge swollen by blood and body fluid.

As illustrated in Figure 5, smooth migration of VEGF into the agar was observed over 72 h, confirming that the PAA/PVP gel swollen by blood or body fluid has the capacity to gradually release growth factors toward the wound surface. The PAA/PVP complex gel functions as a hydrogel network, within which water-soluble macromolecules such as cytokines and growth factors diffuse relatively slowly through hydrogel matrices, allowing them to be retained for extended periods and gradually released over time. This sustained release likely maintains a favorable local concentration of cytokines at the wound interface, which may contribute to the observed rapid and clean healing. In addition to passive retention and release, it is also hypothesized that live blood cells infiltrate into the swollen gel and actively keep secreting cytokines such as VEGF within the gel matrix. These molecules may be retained and subsequently diffuse out. Although this cellular secretion-based mechanism was not quantitatively verified in the present study, it remains a plausible contributor and warrants further investigation in future in vivo studies.

Both PAA and PVP are safe polymers that have been approved and widely used as pharmaceutical and food additives for many years. Since both are synthetic polymers, they pose no risk of viral infection or immunogenicity. The swellable PAA/PVP complex used in this study has previously been confirmed and reported to exhibit minimal cytotoxicity [17]. In this study, microbial-derived hyaluronic acid was incorporated as a reinforcing additive, which is also a well-established, safe polymer material that does not contain animal-derived proteins. As a mixture of these raw materials, the wound dressing developed here is tissue-adhesive, easy to handle, safe, and holds promise as a medical material with wound healing-promoting effects.

In this study, the wound healing effect of the PAA/PVP complex was evaluated using a diabetic skin defect model. Although the results demonstrated enhanced healing in this model, the interactions of the hydrogel with other types of wounds, such as burn injuries or oral wounds, were not examined. However, anecdotal observations from dental practitioners who used a hemostatic material based on the same PAA/PVP composition have suggested potentially accelerated healing in oral applications. While these impressions are encouraging, no quantitative data are currently available. Further studies using various wound models are necessary to fully explore the therapeutic potential of this material across different tissue environments.

In addition to biological performance, the feasibility of large-scale production is a critical consideration for clinical application. Although a product using the same formulation has already been commercialized for dental use, differences in final shape and application site may affect the consistency of gelation and adhesive properties. Further optimization and quality control measures may be necessary to ensure uniform performance across different use cases in large-scale manufacturing.

## 3. Conclusions

In this study, it was demonstrated that a physically crosslinked PAA/PVP complex gel can adhere strongly to wet tissues and maintain a moist wound environment. When applied to skin defects in diabetic mice, this hydrogel significantly accelerated wound closure: wound size decreased to approximately 70–75% by day 4 and further reduced to 17–23% by day 11. Histological evaluation confirmed nearly complete re-epithelialization in the treated wounds, compared to poor regeneration in the controls. Furthermore, in vitro studies indicated that blood-derived cytokines such as VEGF remained within the swollen gel and gradually diffused toward surrounding tissues for over 72 h. These results suggest that the PAA/PVP complex not only provides physical protection, but also actively promotes tissue regeneration, highlighting its potential as a next-generation wound dressing.

However, this study has some limitations. First, while the in vitro cytotoxicity and biodegradability of the PAA/PVP complex have been extensively characterized in previous research, biocompatibility assessments were not conducted within the scope of the present study. Second, although the gel demonstrated efficacy in a diabetic mouse wound model, its performance in other wound types and in human tissues remains to be validated. Additionally, the sustained release of cytokines from the gel was observed, but detailed analyses are required to elucidate the underlying mechanisms. Finally, the long-term stability of the hydrogel when applied to wound surfaces has not yet been evaluated. These aspects should be addressed in future studies to fully assess the clinical potential of the PAA/PVP complex.

## 4. Materials and Methods

### 4.1. Mice

Female BKS.Cg-+Leprdb/+Leprdb/Jcl mice and normal female BALB/c mice were purchased from CLEA Japan, Inc. (Tokyo, Japan) and SLC (Shizuoka, Japan), respectively. All animal experiments were conducted in accordance with the protocol approved by the Tokyo Medical University Animal Committee (R6-015).

### 4.2. Materials

PAA for pharmaceutical use (Carbopol 934P NF) and PVP (Kollidon 90F; MW = 320,000 g/mol) were purchased from Kobayashi Perfumery Co., Ltd. (Tokyo, Japan), and BASF Japan Ltd. (Tokyo, Japan), respectively. Sodium hyaluronate (HA) fermented by Streptococcus zooepidemicus (HA-LQH; MW = 1,500,000–2,200,000 g/mol) was obtained from Kewpie Corporation (Tokyo, Japan).

### 4.3. Preparation of PAA/PVP Complex Sponge

A stick-shaped PAA/PVP complex sponge (7 mm × 7 mm × 25 mm) was prepared similarly as described in our previous paper; typically, an aqueous PAA solution (1.5%, 1.2 mL) was added to a silicone mold with a thickness of 7 mm and through-holes of 25 mm length and 7 mm width attached to the polypropylene sheet, and dried up at rt for 24 h to a transparent film. The aqueous solution of PVP and HA (PVP: 1.98%; HA: 0.26%; 1.4 mL) was then added to the PAA film. After incubation at 28 °C for a given time, the swollen PAA/PVP complex was frozen at −60 °C, and then freeze-dried with a freeze-dryer CS-55 (SAKUMA Seisakusho Co., Ltd., Tokyo, Japan). For animal wound healing tests, a square-shaped PAA/PVP complex sponge (14 mm × 14 mm) containing 11.8 mg of PAA, 18.2 mg of PVP, and 2.36 mg of HA was prepared similarly, using a silicone mold with a thickness of 5 mm.

### 4.4. Measurement of the Mechanical Strength of the PAA/PVP Sponge

The mechanical strength of the stick-shaped PAA/PVP sponge was evaluated using a three-point bending test conducted at 25 °C and 27% relative humidity. A 7 mm × 7 mm × 25 mm, the PAA/PVP complex sponge was set in a tensile testing machine (ZTA-50N digital force gauge and MX2-500N test stand (Imada Co., Ltd., Aichi, Japan)) in various orientations and compressed at a speed of 50 mm/min. The force at which the sponge collapsed was recorded.

### 4.5. Measurement of Pull-Off Adhesion Strength of the PAA/PVP Sponge

A PAA/PVP complex sponge (7 mm × 7 mm × 25 mm) was cut perpendicularly to its long axis near the center. The resulting fragment was fixed in the upper clamp with the cut surface facing downward. A synthetic leather piece (10 mm × 10 mm) was attached to the upper surface of an aluminum block. A given amount of water was added to the synthetic leather piece, and the upper support holding the PAA/PVP complex was lowered at a speed of 30 mm/min until a compression force of 2 N was reached. The support was stopped, and the pressing force was maintained at 2 N for 10 s. The upper support with the PAA/PVP complex was then raised at a speed of 15 mm/min, and the force at which the complex detached or collapsed was recorded.

### 4.6. Wound-Healing Acceleration by the PAA/PVP Complex Sponge

To evaluate the wound-healing efficacy of the PAA/PVP complex sponge, a diabetic wound model was used. Female BKS.Cg-+Leprdb/+Leprdb/Jcl mice (7 weeks old) were randomly assigned into three groups (*n* = 3 mice per group). Under general anesthesia with a combination of medetomidine hydrochloride (0.3 mg/kg), butorphanol tartrate (5.0 mg/kg), and midazolam (4.0 mg/kg), the dorsal hair was shaved and the skin was disinfected. Two circular full-thickness excisional wounds (8 mm in diameter) were created symmetrically on the dorsal skin using a sterile 8 mm biopsy trepan. A square-shaped PAA/PVP sponge (14 mm × 14 mm) prepared as above was applied to both wound sites immediately after wounding. The sponge was removed either 1 or 3 days after application depending on the group. Wound size was measured from photographs using ImageJ software (Version 1.53s, NIH, Bethesda, MD, USA) on days 0, 1, 2, 3, 4, 7, 9, and 11 post-injury. At 11 days after wounding, mice were euthanized and tissue samples including the wound area and surrounding skin were collected, fixed in 10% neutral-buffered formalin, embedded in paraffin, and sectioned. The resulting tissue sections were subjected to hematoxylin and eosin (HE) staining for histological analysis of epithelial regeneration. Histological evaluation was performed using an optical microscope (CKX53, Olympus Corporation, Tokyo, Japan).

### 4.7. Slow Release of Growth Factors from the PAA/PVP Complex Sponge

The circular wounds (8 mm in diameter) were created on the back of normal BALB/c mice (7 weeks old) under anesthesia, as described above. A 1 cm × 1 cm square PAA/PVP sponge, composed of PAA (6 mg), PVP (9.24 mg), and HA (1.2 mg), was applied to the wound overnight. The PAA/PVP complex gel swollen by the blood or body fluid was then peeled off and placed on the surface of 1% agarose gel (1 mL) prepared in PBS. At a given time, the PAA/PVP gel was transferred onto the next agarose gel. Each agarose gel was crushed into a paste, and was mixed with 2 mL of PBS. It was centrifuged at 3000× *g* for 15 min at 4 °C, and the supernatant was collected. The concentration of VEGF in the supernatant was measured using an ELISA kit (Proteintech Group, Tokyo, Japan).

## Data Availability

The original contributions presented in this study are included in the article, and further inquiries can be directed to the corresponding authors.

## References

[1] Dhivya S., Padma V.V., Santhini E. (2015). Wound dressings—A review. BioMedicine.

[2] Simões D., Miguel S.P., Ribeiro M.P., Coutinho P., Mendonça A.G., Correia I.J. (2018). Recent advances on antimicrobial wound dressing: A review. Eur. J. Pharm. Biopharm..

[3] Goldenberg M.S. (1996). Wound care management: Proper protocol differs from athletic trainers’ perceptions. J. Athl. Train..

[4] Behrens A.M., Sikorski M.J., Kofinas P. (2014). Hemostatic strategies for traumatic and surgical bleeding. J. Biomed. Mater. Res. A.

[5] Reinke J.M., Sorg H. (2012). Wound repair and regeneration. Eur. Surg. Res..

[6] Koh T.J., DiPietro L.A. (2011). Inflammation and Wound Healing: The Role of the Macrophage. Expert Rev. Mol. Med..

[7] Barrientos S., Stojadinovic O., Golinko M.S., Brem H., Tomic-Canic M. (2008). Growth factors and cytokines in wound healing. Wound Repair Regen..

[8] Gharbia F.Z., Abouhashem A.S., Moqidem Y.A., Elbaz A.A., Abdellatif A., Singh K., Azzazy H.M. (2023). Adult skin fibroblast state change in murine wound healing. Sci. Rep..

[9] Eaglstein W.H. (2001). Moist wound healing with occlusive dressings: A clinical focus. Dermatol. Surg..

[10] Winter G. (1962). Formation of the Scab and the Rate of Epithelization of Superficial Wounds in the Skin of the Young Domestic Pig. Nature.

[11] Winter G.D. (1963). Effect of air exposure and occlusion on experimental human skin wounds. Nature.

[12] Zhang W., Liu L., Cheng H., Zhu J., Li X., Ye S., Li X. (2024). Hydrogel-Based Dressings Designed to Facilitate Wound Healing. Mater. Adv..

[13] Gounden V., Singh M. (2024). Hydrogels and Wound Healing: Current and Future Prospects. Gels.

[14] Jiang Y., Zhang X., Zhang W., Wang M., Yan L., Wang K., Han L., Lu X. (2022). Infant Skin Friendly Adhesive Hydrogel Patch Activated at Body Temperature for Bioelectronics Securing and Diabetic Wound Healing. ACS Nano.

[15] Liu K., Zhang C., Chang R., He Y., Guan F., Yao M. (2023). Ultra-Stretchable, Tissue-Adhesive, Shape-Adaptive, Self-Healing, on-Demand Removable Hydrogel Dressings with Multiple Functions for Infected Wound Healing in Regions of High Mobility. Acta Biomater..

[16] Ito T., Eriguchi M., Koyama Y. (2015). Bioabsorbable bioadhesive hydrogel comprising poly (acrylic acid) and poly (vinylpyrrolidone) for adhesion barrier and hemostatic device. MRS Commun..

[17] Ito T., Otani N., Fujii K., Mori K., Eriguchi M., Koyama Y. (2020). Bioadhesive and biodissolvable hydrogels consisting of water-swellable poly (acrylic acid)/poly (vinylpyrrolidone) complexes. J. Biomed. Mater. Res. B Appl. Biomater..

[18] Ito T., Yamaguchi S., Soga D., Ueda K., Yoshimoto T., Koyama Y. (2022). Water-Absorbing bioadhesive poly (acrylic acid)/polyvinylpyrrolidone complex sponge for hemostatic agents. Bioengineering.

[19] Bimendina L.A., Bekturov E.A., Roganov V.V. (1976). Poly (Acrylic Acid)-Poly (Vinyl Pyrrolidone) Complexes in Solutions. Chem. Pap..

[20] Vasheghani Farahani B., Hosseinpour Rajabi F., Ahmadi M.H., Zenooz N. (2012). Qualitative Investigation on Some H-Bonded Interpolymer Complexes by Determination of Thermodynamic Parameters. J. Mex. Chem. Soc..

[21] Whitehead F., Giampieri S., Graham T., Grocott P. (2017). Identifying, managing and preventing skin maceration: A rapid review of the clinical evidence. J. Wound Care.

